# The basal translation rate of authentic HIV-1 RNA is regulated by 5’UTR nt-pairings at junction of R and U5

**DOI:** 10.1038/s41598-017-06883-9

**Published:** 2017-07-31

**Authors:** I. Boeras, B. Seufzer, S. Brady, A. Rendahl, X. Heng, K. Boris-Lawrie

**Affiliations:** 1 University of Minnesota, Department of Veterinary and Biomedical Sciences, 1971 Commonwealth, Saint Paul, MN 55108 USA; 2 University of Missouri, Department of Biochemistry, 503 S. College Ave, Columbia, MO 65211 USA

## Abstract

The paradigm protein synthesis rate is regulated by structural complexity of the 5′untranslated region (UTR) derives from bacterial and other riboswitches. In-solution, HIV-1 5′UTR forms two interchangeable long-range nucleotide (nt) -pairings, one sequesters the gag start codon promoting dimerization while the other sequesters the dimer initiation signal preventing dimerization. While the effect of these nt-pairings on dimerization and packaging has been documented their effect on authentic HIV translation in cellulo has remained elusive until now. HIV^NL4-3^ 5′UTR substitutions were designed to individually stabilize the dimer-prone or monomer-prone conformations, validated in-solution, and introduced to molecular clones. The effect of 5′UTR conformation on ribosome loading to HIV unspliced RNA and rate of Gag polypeptide synthesis was quantified in cellulo. Monomer- and dimer-prone 5′UTRs displayed equivalent, basal rate of translation. Gain-of-function substitution U103, in conjunction with previously defined nt-pairings that reorient AUG to flexible nt-pairing, significantly activated the translation rate, indicating the basal translation rate is under positive selection. The observed translation up-mutation focuses attention to nt-pairings at the junction of R and U5, a poorly characterized structure upstream of the characterized HIV riboswitch and demonstrates the basal translation rate of authentic HIV RNA is regulated independently of monomer:dimer equilibrium of the 5′UTR.

## Introduction

RNA viruses require structural information conserved in noncoding sequences to carry-out critical events in their biogenesis. The 5′ untranslated region (UTR) of retroviruses is a bevy of cis-acting information that is communicated through various higher order structures whose primary sequences overlap. Genetic, biophysical and virological studies have dissected which sequences are essential for early and late viral activities. For instance, HIV-1 5′UTR has activity as mRNA template translated to viral structural proteins, and as dimerized genomic ribonucleoprotein (RNP) packaged into the virion^1, 2^. This dual utility of identical primary sequence is attributable to switching between higher-order conformations of the 5′UTR by a process that is poorly reconciled.

A major accomplishment over the past twenty-years is the genetic and structural basis retroviral noncoding sequences dimerize and foster packaged diploid genomic RNA^3–17^. Long-distance pairings between the unique 5′ (U5) nucleotides (nts) of HIV primary RNA and downstream nts around the gag start codon (AUG), function in common purpose to orient a palindromic dimer initiation sequence (DIS) for intermolecular dimerization. The higher order, dimeric conformation induced by U5:AUG nt - pairings facilitates packaging of gRNP to nascent virions^3–5, 7, 10, 15, 18^.

Ironically, an alternative conformation of HIV 5′UTR is induced by reorientation of U5 nts to pair with DIS nts; this conformation abrogates intermolecular dimerization and downregulates selective packaging of virion precursor RNA^10^. Documented by nuclear magnetic resonance (NMR), the U5:DIS pairings induce rearrangement of nts around AUG into a relatively flexible stem loop that is characteristic of a monomer conformation in-solution^10^. The NMR indicates U5:DIS pairings may also reorient higher order structure upstream of U5, although those details remain to be characterized^10^.

Given that the U5:DIS pairings: i) reorient AUG nts into flexible conformation; ii) downregulate 5′UTR dimerization; and iii) reduce gRNP packaged to virions, the monomer-prone 5′UTR in authentic HIV RNA was postulated to be the RNA template for translation to Gag and Gag-Pol^3, 10^. Designated the HIV riboswitch or RNA switch model, this hypothesis invoked principles established for bacterial riboswitches, in which translation is attenuated by specific conformations in noncoding RNA induced by ligand binding^3, 4, 6, 10, 18–20^. The corollary components of an HIV RNA switch are 5′UTR binding to Gag structural protein via the nucleocapsid domain, NC. NC exhibits preferential affinity to the 5′UTR experiencing U5:AUG pairings^7, 21^. Hence, the HIV RNA switch model construed U5:DIS pairings manifest the mRNA template for Gag synthesis, while U5:AUG pairings attenuate translation and activate dimerization and packaging of diploid gRNP.

Resonating with the HIV RNA switch model are observations of murine leukemia virus (MLV), in which genome-length RNA segregated into two physically distinct pools^2, 22, 23^. Using actinomycin D to downregulate gene transcription revealed MLV transcripts were exclusively downregulated as template for translation, but not a substrate for intermolecular dimerization and gRNP biogenesis^22, 23^. These mutually exclusive fates of MLV nascent transcripts were attributed to alternative conformations of the 5′UTR induced by nuclear RNA binding proteins and potentially Gag^24^. Since HIV Rev/RRE activity is downregulated by actinomycin D, alternative approaches, including use of translation inhibitors, were employed to evaluate the fate of HIV RNA. Studies documented HIV RNAs packaged to virions did not require prior usage as mRNA template, positing nascent HIV experiences mutually exclusive fates similar to MLV transcripts^25–29^.

Alternative approaches to address the HIV RNA switch hypothesis demonstrated sequence changes in HIV gag open reading frame that eliminated synthesis of Gag diminished the packaging efficiency of genomic RNA^30–32^. The authors concluded nascent HIV RNA exhibiting monomer-prone 5′UTR conformation templates Gag synthesis, and binding by the nascent Gag polypeptide induces its intramolecular conversion and utility as substrate to be packaged gRNP^3, 30–32^. This intramolecular conversion occurs in cis- and switches U5:DIS pairings to release DIS and sequester the gag start codon by U5:AUG pairings. This scenario, also designated the cis-packaging model^33^, resonates with results of experiments on avian sarcoma virus elucidating a single functional pool of the retroviral transcript used for translation and packaging^2, 34, 35^. Whether or not a conformational change exists on the same HIV RNA molecule or a distinct molecule remains controversial, and has been challenging to address in cell-based assays.

The central tenet of the HIV RNA switch model, translation attenuation by dimer-prone 5′UTR, has been abundantly addressed in context of synthetic transcripts and reporter RNAs. Substitution mutations were designed to favor the thermodynamic stability of either monomer- or dimer-prone RNA and evidence for translation attenuation was ascertained. Synthetic transcripts that were pre-dimerized demonstrated a dearth of *in vitro* translation activity^4^, hence supporting the prediction that U5:AUG conformation attenuates translation. Also, introduction of HIV 5′UTR sequences to synthetic reporter RNAs was sufficient to diminish reporter protein output^36^. By comparison, thermal denaturation or gross deletions that perturbed the U5:AUG conformer upregulated ^35^S-cysteine incorporation to *in vitro* translated Gag polypeptide^37^.

Cell-based studies of the Luciferase reporter RNAs documented equivocal translation activity between native HIV LAI 5′UTR, which is prone to dimerize in-solution and variant 5′UTRs favoring monomer conformation^3^. Results in a follow up-study identified issues in the post-transcriptional expression of select 5′UTR-luc reporter RNAs, which called into question the conclusions made previously^38^.

A potential caveat to the HIV RNA switch model comes from studies of the HIV 5′UTR in context of bicistronic reporter RNAs. Herein, HIV 5′UTR sequences were placed in the intergenic position of vector RNAs and engendered equivocal reporter gene activity, indicative of detectable internal ribosome entry and cap-independent translation^39–42^. Taken together and despite extensive reporter RNA analyses *in vitro* and in cell-based assays, a significant knowledge gap exists of whether or not dimer-prone (U5:AUG) 5′UTR attenuates HIV translation or if monomer-prone (U5:DIS) conformation is necessary to authentic viral protein synthesis. This conundrum engenders impetus to revisit the HIV RNA switch model and will require sensitive measurements of translation rate on authentic HIV genome length RNAs.

Herein, HIV 5′UTR substitutions favoring U5:AUG pairings or U5:DIS pairings, which are diagnostic of the dimer-prone or the monomeric 5′UTR, respectively, were introduced to HIV^NL4-3^ molecular clones. The molecular clones were introduced to cells that were subjected to kinetic measurement of Gag translation and quantitation of steady state and polysomal RNA. The molecular determinants of Gag translation rate in authentic HIV RNA were characterized for the first time.

## Results

### HIV 5′UTR substitutions affect dimerization *in vitro*

**Figure 1:**
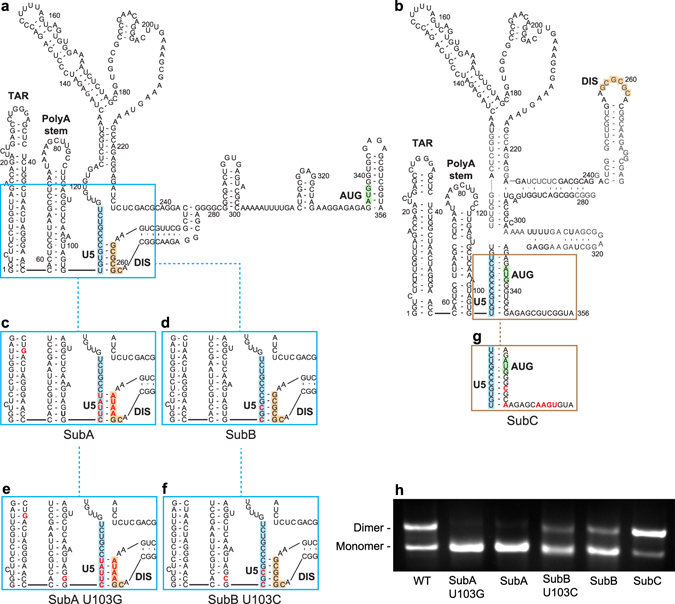
Models of the HIV^NL4-3^ 5′UTR conformations with select nt substitutions to alter monomer-dimer equilibrium. Models of HIV^NL4-3^ 5′UTR (+1 to 356) (**a**) monomer and (**b**) dimer structures from Lu *et al*.^10^. Labels designate: TAR, PolyA stem, U5 (blue), DIS (orange), AUG (green), and nt substitutions (red). Boxes highlight sequences proximal and distal to U5 involved in monomer (blue) or dimer (brown) conformations. (**c** and **d**) SubA and SubB contain nt changes that favor U5:DIS base-pairing and destabilize U5:AUG base pairing. (**e** and **f**) Additional U103 substitutions destabilize the bottom stem of PolyA in SubA U103G and SubB U103C. (**g**) SubC contains nt substitutions that favor U5:AUG base-pairing and maintain amino acid conservation without rare codon bias. (**h**) Native agarose gel assay shows 5′UTR conformations *in vitro* at equilibrium. WT 5′UTR migrated as a mixture of monomer and dimer. SubC mainly migrated as dimer, whereas each of the other 5′UTR variants mainly migrated as monomer.

While high-resolution structure of the dimeric HIV 5′UTR is available, only a portion of the monomeric HIV 5′UTR has been solved^10^. As summarized in Fig. 1a, NMR of monomeric RNA detects long-range pairings between U5:DIS nts and localized pairings of nts around AUG in a relatively flexible stem loop. *In vitro* results posit U103 and G104 are at the bottom of the loosely paired PolyA stem, while U105 participates in the U5:DIS or U5:AUG interactions^43^. The NMR of dimer-prone 5′UTR detects U5 nts in long-range pairings with nts around AUG, instead of DIS (Fig. 1b)^7, 10^.

Building on the extensive NMR structural studies with synthetic HIV transcripts (+1 to +356), nt substitutions were designed proximal and distal to U5 that increased stability of U5:DIS nt - pairings. As previously probed by NMR, SubA stabilizes the U5:DIS nt - pairings, (Fig. 1c) and SubB U105,107 C are sufficient to stabilize U5:DIS and destabilize U5:AUG (Fig. 1d). The U103 substitutions in SubA U103G and SubB U103C (Fig. 1e and f) conceivably destabilize the bottom stem of PolyA. By comparison, NMR characterization of SubC demonstrated the substitutions stabilize the U5:AUG interaction that is characteristic of the dimer-prone 5′UTR that directs efficient packaging of diploid genomic RNA (Fig. 1g)^7, 10^. Carried over from the prior *in vitro* studies was a naturally occurring A46G substitution that was instrumental to diminish this adenine resonance in NMR studies without affecting structure^10^.

The synthetic WT and variant 5′UTRs were incubated in physiological ionic strength buffer and subjected to native gel electrophoresis. As shown in Fig. 1h, WT HIV^NL4-3^ 5′UTR exhibited two molecular conformations designated monomer and dimer. Stabilizing U5:DIS pairing with or without the U103 substitution (SubA, SubB, SubA U103G, and SubB U103C) changed its mobility to the lower molecular weight monomer conformation (Fig. 1h). SubC favored formation of the higher molecular weight dimer conformer (Fig. 1h). The assay demonstrated the monomer: dimer equilibrium characteristic of native HIV 5′UTR in these conditions was altered by these nt substitutions.

### Molecular clones having 5′UTR substitutions maintained expression of the three classes of HIV transcripts

Next, the substitutions were introduced to HIV^NL4-3^ molecular clones and HIV RNA expression was evaluated in transfected cells. Twenty-four h post-transfection, HEK293 cells were harvested, RNA preparations were extracted, treated with DNase, and subjected to reverse transcription (RT) with random primers and the quantitative PCR (qPCR) with HIV gene-specific primers. The accumulation of unspliced, singly spliced, and multiply spliced HIV RNAs was similar between parental HIV^NL4-3^ and derivatives. As compiled in Table 1, similar levels of these HIV RNA species were observed, indicating expression of the HIV RNA remained similar to WT. Since the nt changes produced no detectable change in steady state of the singly spliced, multiply spliced, or unspliced HIV transcripts, we concluded the 5′UTR substitutions and structural changes did not disrupt post-transcriptional processing of the HIV primary RNA and the molecular clones were suitable tools to investigate the impact of altered 5′UTR conformations in cell-based assays.

**Table 1:** 5′UTR substitutions do not change steady state HIV transcript levels^a^.

5′UTR	HIV transcript (Relative units^b^)			
	Unspliced	Singly spliced	Multiply spliced	Sum
WT	4.3 ± 0.3	4.1 ± 0.2	4.7 ± 0.1	13.1
SubA	4.7 ± 0.1	4.5 ± 0.1	4.9 ± 0.5	14.1
SubB	4.5 ± 0.3	4.4 ± 0.1	4.9 ± 0.1	13.8
SubC	4.3 ± 0.1	4.1 ± 0.1	4.8 ± 0.1	13.2
SubA U103G	4.5 ± 0.1	4.2 ± 0.1	4.7 ± 0.5	13.4
SubB U103C	4.3 ± 0.3	4.3 ± 0.1	4.8 ± 0.1	13.4

^a^Table represents average of three independent experiments ± standard deviation. HEK293 cells were transfected with WT HIV^NL4-3^ or indicated molecular clone, incubated for 24 h and cell lysate was collected and RNA was extracted. Random primers were used to generate cDNA and qPCR was performed using HIV-specific primers.

^b^Relative units = 1/cycle threshold × 100.

### Translation rate increased in select monomer-prone 5′UTRs

To compare translation activity of the 5′UTRs in context of authentic viral RNA, a sensitive, cell-based kinetic assay was developed, as summarized in Fig. S1. Cells transfected with each molecular clone were briefly incubated in medium depleted of cysteine and methionine, halting new protein synthesis, while allowing for ribosome runoff and the loading of preinitiation complexes to activated mRNA templates. Next, the cultures were supplemented with ^35^S-methionine and cysteine (^35^S-cys/met) to support new protein synthesis from activated mRNA templates. Newly synthesized polypeptides were isolated by immunoprecipitation (IP) with specific antisera, subjected to SDS-PAGE and phosphorimaged to quantify ^35^S-incorporation to specific polypeptide. The ^35^S-cys/met incorporation into Gag measured the translation activity of HIV 5′UTRs.

**Figure 2:**
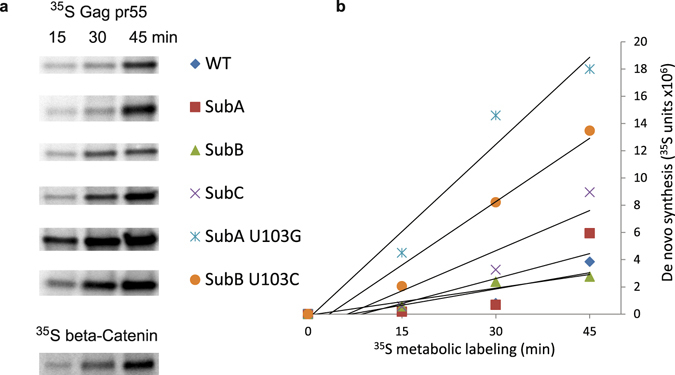
The rate of de novo Gag synthesis from dimer-prone 5′UTR is similar to some, but not all, monomer 5′UTRs. A representative kinetic experiment using the metabolic labeling approach described in Figure S1 is presented. Rate of gag RNA translation was similar between HIV^NL4-3^ WT and SubC dimer-prone 5′UTRs and monomer-prone SubA and SubB, yet significantly increased by U103 substitutions (SubA U103G and SubB U103C) at PolyA stem. (**a**) SDS-PAGE and phosphorimage of ^35^S-Gag precursor (pr55 Gag) and beta-Catenin loading control. Full-length gels are provided in Figure S2. (**b**) Quantification of ^35^S-Gag polypeptides measured by ImageQuant in this representative experiment with trendlines reflecting differences in translation rate.

Control experiments established new polypeptide synthesis was reliably detectable within 15 min incubation and de novo synthesis of Gag and loading control beta-Catenin increased during successive 15-min intervals (Fig. 2 and Fig. S2). Different activity of mRNA templates was resolvable within 45 min and IP controls indicated that by 60 min, the concentration of Gag-specific antiserum began to limit sensitivity of the assay (data not shown). Sufficient statistical power was achieved by harvesting samples at three 15-min intervals in multiple replicate experiments with the molecular clones.

Gag synthesis during the successive time points was similar between WT and the SubA, SubB, SubC 5′UTRs, indicating the translation rate of these mRNAs remained similar, despite different stabilities of U5:DIS pairings. By contrast, SubA U103G and SubB U103C 5′UTRs significantly increased ^35^S-incorporation to Gag, indicating these 5′UTR conformations increased the rate of translation (Fig. 2a and b).

**Figure 3:**
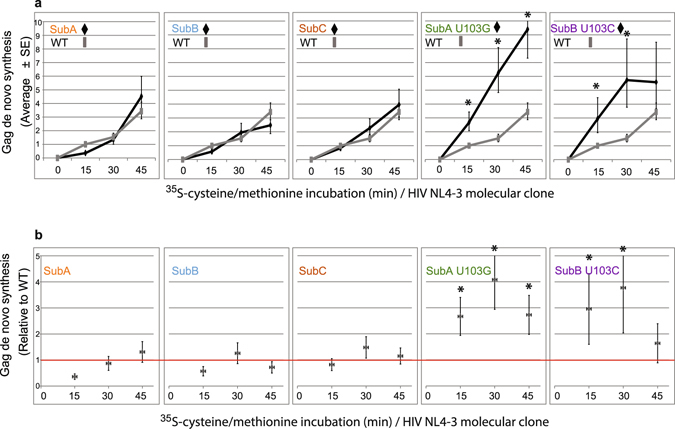
Addition of U103 substitution to monomer 5′UTRs significantly increased translation rate. Statistical analysis of results of five independent translation rate experiments (representative in Fig. 2) with each molecular clone by a linear mixed model. Statistically significant differences relative to WT (p < 0.05) (astericks) were validated in the rate of polypeptide synthesis between WT, SubA U103G and SubB U103C, but not SubA, SubB, SubC. (**a**) Average ratio (±standard error) of de novo Gag synthesis at each time relative to WT at time 15. Separate plots present each molecular clone (black line) relative to same WT (grey line). (**b**) Average fold difference (±standard error) at each time interval in de novo Gag synthesis relative to WT. Red line, no difference from WT.

Results of five independent experiments were fitted to a linear mixed model and the estimated values at each time point were normalized to WT at the 15-min interval. In Fig. 3a, the translation rate of each molecular clone (black line) was compared to WT (gray line). Pairwise comparisons revealed no statistical difference at the 0.05 significance level in translation rate between WT (gray line) and SubA, SubB and SubC (black lines) (Fig. 3a). Statistically significant differences were observed between WT and SubA U103G and SubB U103C (Fig. 3a).

More statistical comparisons evaluated differences in ^35^S-labeled Gag between each timepoint. Results determined the differences observed between WT and SubA U103G and SubB U103C were significant at 15 and 30 min time points; exception was experienced by SubB U103C at the 45 min time point (Fig. 3b). The difference in rate peaked at 30 min, when 4-fold increase was observed for SubA U103G and SubB U103C compared to WT (Fig. 3b). On average, their translation rates were 3- and 2.6-fold greater than WT, respectively (Table S1). Parallel analysis of ^35^S-cys/met incorporation to beta-Catenin in the lysates validated translation activity of beta-Catenin mRNA remained equivalent in paired samples (Table S1).

In summary, the sensitivity of this metabolic labeling assay was sufficient to measure the basal translation rate of the HIV genome length RNA. Statistical analysis documented similar HIV translation rates between dimer-prone 5′UTRs (WT and SubC) and monomer-prone 5′UTRs that were identical except for destabilized U5:AUG pairings and nonsequestered AUG (SubA and SubB). The results demonstrate U5:AUG pairings were not sufficient to attenuate Gag translation, as postulated in the HIV RNA switch model^3, 10^. Translation rate was significantly increased in response to an upstream U103 substitution (Figs 2 and 3 and Table S1), positing a gain-of-function role for U103 substitution in HIV RNA translation.

### HIV polysomes were activated by select monomer-prone 5′UTRs

**Figure 4:**
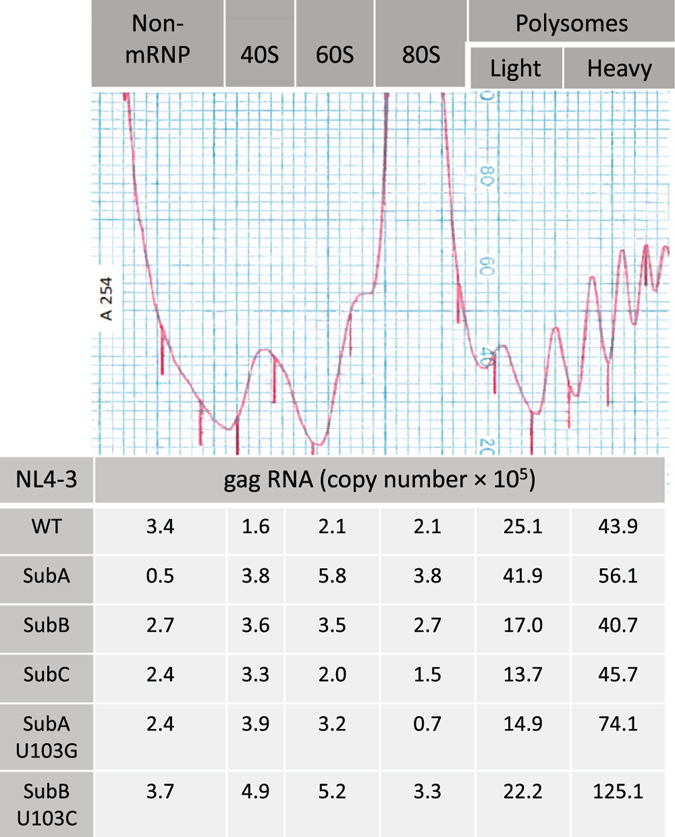
Quantitative analysis of HIV polysomes documents equivalent translation activity of dimer- and monomer-prone RNA and up-regulation by addition of U103 substitution. Representative ribosomal profile with labeling of RNP populations and distribution of gag RNA quantified by RT-qPCR. Results were similar between clones, with exception that SubA U103G and SubB U103C significantly increased HIV polysomes. Standard curves were used to measure gag RNA copy number in these samples.

**Figure 5:**
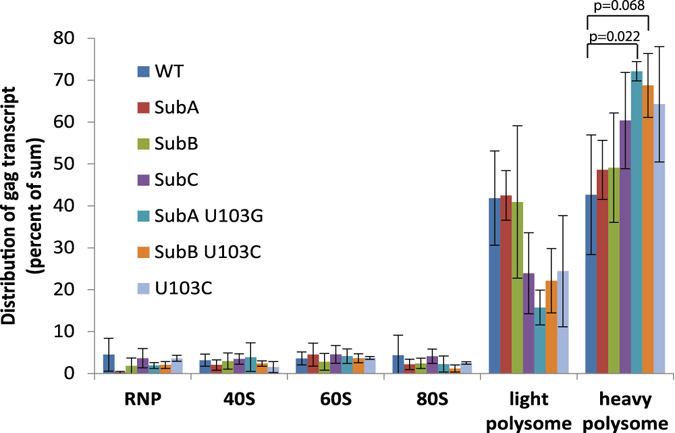
Addition of U103 substitution to monomer 5′UTRs significantly increased HIV polysomes. Statistical analysis of replicate ribosome profile experiments with each molecular clone measuring distribution of gag RNA by RT-qPCR. The percentage in each RNP is the average ± standard error of three independent experiments and statistically significant differences relative to WT denoted in brackets.

Differences in mRNA translation activity are measurable by quantifying RNA accumulation on polysomes. To measure HIV polysomes, cytoplasmic lysates of the HIV expressing cells were isolated, RNPs were fractionated over 10–50% continuous sucrose gradients monitored at UV^254^ and transcripts in each fraction were collected using Trizol. Guided by the profile of ribosomal RNA (UV^254^), the fractions were collected into designated groups: the low density non-ribosomal RNPs, and 40 S, 60 S, 80 S RNPs, light polysomes (two and three ribosomes) and heavy polysomes (four or more ribosomes) (Fig. 4). A representative experiment shows the differences between molecular clones in HIV gag transcript abundance across the gradients (Fig. 4). Ribosomal RNA profiles were equivalent in multiple replicate 24 h transfection experiments (Fig. S3). The amount of gag RNA in each RNP in the multiple experiments was measured by RT-qPCR and statistical analysis identified WT, SubA, SubB, and SubC RNAs exhibited similar distribution across the gradients (Fig. 5). For SubA U103G and SubB U103C, the heavy polysome accumulation was significantly increased (p ≤ 0.022 and p ≤ 0.068, respectively) (Fig. 5). This distinction between SubA U103G and SubB U103C and the other 5′UTRs (p ≥ 5.0) recapitulated their increased translation rate in the kinetic assay. An explanation for these results is increased recruitment of ribosomes to SubA U103G and SubB U103C 5′UTRs.

To discern whether or not substitution of U103 alone was sufficient to increase HIV polysomes, a sole U103C substitution was introduced to HIV^NL4-3^ and synthetic transcripts were subjected to the gel mobility shift assay. Compared to WT, U103C exhibited preference for monomer conformation, indicating this single nt upstream of U5 was sufficient to destabilize dimer conformation and presumably the U5:AUG pairings (Fig. S4). Next the polysome accumulation was compared between HIV^NL4-3^U103C and WT, revealing a modest increase of U103C RNA in heavy polysomes that did not reach statistical significance (Fig. 5). Moreover, U103C was modestly diminished relative to SubAU103G and SubBU103C. We concluded the U103C substitution of the HIV 5′UTR, which was sufficient to destabilize U5:AUG in-solution, produced an intermediate phenotype between WT and the substitutions directly disrupting U5:AUG. In summary, U5:DIS and U5:AUG pairings supported similar translation activity (designated basal) demonstrating HIV translation is not attenuated by dimer-prone 5′UTR. Rather, destabilized nt pairing at the bottom of the PolyA stem in conjunction with U5:DIS pairings (and destabilized U5:AUG) were necessary to significantly increase HIV translation rate, demonstrating a critical role in translation control contributed by 5′ proximal ~100 nt of HIV.

## Discussion

Sequestering AUG by long-range U5:AUG nt-nt interactions does not attenuate translation of HIV RNA. Our results of kinetic experiments and quantification of HIV polysomes demonstrated that specifically stabilizing U5:DIS pairings, and therefore reorienting AUG to a flexible stem, is insufficient to increase translation activity of 5′ UTR in authentic genome-length viral RNA. That monomeric and dimer-prone 5′UTRs exhibit similar translation rate indicates HIV translation regulation is not simply due to the occlusion of the gag AUG in dimer-prone 5′UTRs.

Unexpectedly, U103 substitution is sufficient to shift monomer:dimer equilibrium toward monomer conformation in-solution. This perturbation of nt-pairings at the base of the PolyA stem (nt 59–103) can be explained by shifted register of localized nt-nt pairings. The U103 substitution modestly increased HIV translation activity alone, but significantly enhanced translation when in combination with SubA or SubB substitutions that release U5:AUG nt-pairings, implicating a synergistic effect on 5′UTR structure. Recently a similar structural perturbation at the base of PolyA stem was documented and proposed to be a consequence of heterogeneity in the HIV transcription start site^43, 44^. The translation activity of U103 perturbation draws attention to the gap in knowledge of three-dimensional structure spanning TAR and PolyA stem. Historically, this segment of the HIV RNA, which corresponds to junction of R and U5 regions of the 5′ long terminal repeat (LTR), has proven unwieldy in otherwise highly informative biophysical assays, including FRET-based analysis by Lever and colleagues^15^.

In refocusing attention to the role of RU5, this segment of HIV, HTLV-1 and several other retroviruses has been shown to activate polysome loading and increase Gag protein synthesis in conjunction with DHX9/RNA helicase A, and potentially other cellular RNA binding proteins^45–49^. Studies comparing HIV-1 and HIV-2 translation activity revealed HIV-2 TAR structure is more inhibitory than HIV-1^50^. Given unprecedented translational activity uncovered at the junction of HIV-1 R and U5, structural parameters of this segment of the 5′UTR, which has evaded characterization in other solved HIV RNA structures, will be critical to identify.

In closing, our study documented complexity of HIV translational control is intertwined with 5′UTR conformations broader than monomer:dimer equilibrium. We are now asking the questions: how does 5′UTR structure influence interaction with the nuclear cap binding complex and its retention on HIV RNA during downregulation of eIF4E^51^; what is the structural consequence to adenine methylation within the 5′UTR^52–54^ or the trimethylation of the 5-terminal 7-methylguanosine cap^55^. Finally, mechanistic studies are warranted to characterize the unprecedented role of DHX9/RHA in translational control of the 5′-proximal residues of HIV RNA situated between TAR and the PolyA stem^45–47^.

## Materials and Methods

### *In vitro* transcripts and RNA dimerization assay

Template DNAs contained a T7 promoter adjacent to HIV^NL4-3^ 5′UTR residues (+1 to +356) in pUC19 and sequences were introduced by site-directed mutagenesis (Agilent). *In vitro* transcription reactions contained T7 RNA polymerase, PCR amplified template DNA (1.5 µg) in 30.8 mM Tris-HCl, 6 mM MgCl_2_•6H_2_O, 1 mM spermidine, 5 mM DTT, 1.1 µM Triton X-100, and NTPs for 120 min at 37 °C^56^. Reactions were loaded to 6% denaturing urea gels (SequaGel) and the transcripts were eluted by Elutrap Electroelution (Whatman). The RNA dimerization assay used aliquots of 0.65 µM RNA in 10 mM Tris-HCl (pH 7.5) in boiling water for 3 min and then transferred to ice. Samples were adjusted to 0.5 µM in 1x physiological ionic strength buffer (140 mM KCl, 10 mM NaCl, 1 mM MgCl_2_) and incubated at 37 °C for 24 h. Electrophoresis at 4 °C used 2.5% native agarose gels prepared in TB buffer (44.5 mM Tris-boric acid, pH 7.5) and RNA was visualized by ethidium bromide staining.

### Cell lines and transfection

HEK293 cells were maintained in EMEM (ATCC) plus 10% FBS (Gibco) and 1 × Anti-Anti (Gibco) and seeded in 12-well plates for transfections (2 × 10^5^ per well). After overnight culture, cells were incubated with 500 ng pNL4-3 or derivatives and 1.5 µl XtremeGene (Invitrogen) in OptiMEM (100 µl). The 5′UTR sequences of SubA, SubB, SubC, SubA U103G, and SubB U103C were generated by site-directed mutagenesis (Stratagene) and verified by plasmid restriction and sequencing. After 24 h incubation, cells were lysed in 250 µl ice-cold RIPA buffer (50 mM Tris, 150 mM NaCl, 1% NP40, 0.25% deoxycholic acid, 1 mM EDTA).

### RNA extraction and quantification

Cell lysate was mixed with Trizol (Invitrogen) and RNA isolated per manufacturer instructions and treated with (2 units) TurboDNAse (Ambion) for 60 min at 37 °C, followed by 5 µl DNAse inactivation reagent (Ambion). Samples were applied to RNeasy Clean-up columns (Qiagen) and RNA was eluted in DEPC-treated sterile water. Five-hundred ng of RNA per sample was incubated with 2 µl random hexamers (Invitrogen) and 4 units Omniscript RT (Qiagen) in 20 µl reactions for 60 min at 37 °C. Typically, 2 µl of each sample was subjected to real-time quantitative PCR with BioRad SYBR Green and HIV NL4-3 gene-specific primers: gag/unspliced KB1614 GTAAGAAAAAGGCACAGCAAGCAGC and KB1615 CATTTGCCCCTGGAGGTTCTG; singly spliced KB2299 GGCGGCGACTGGAAGAAGC and KB2300 CTATGATTACTATGGACCACAC; and doubly spliced KB 2301 GACTCATCAAGTTTCTCTATCAAA and KB2302 AGTCTCTCAAGCGGTGGT.

### Metabolic labeling and immunoprecipitation assays

Transfected cells were cultured in 1 ml cysteine/methionine-free EMEM (Gibco) for 30 min. This medium was replaced with 300 µl EMEM/10% dialyzed fetal bovine serum/1 × L-glutamine (Gibco) supplemented with 100 μCi/ml ^35^S-labeled cysteine/methionine (Perkin Elmer). At 15 min intervals, the medium was decanted and cells were harvested in ice-cold 250 μl RIPA buffer by gentle pipetting, and soluble proteins were collected by the centrifugation step. The entire sample was mixed with 20 µl pretreated Dynabeads and incubated at 4 °C for 3 h on a rotating platform. The pretreated Dynabeads were conjugated to an antiserum as described^57^. We used 2 µl HIV Gag 24–4 (NIH AIDS Reagent Program)^58, 59^ or 2 µl beta-Catenin (Sigma) antiserum for each immunoprecipitation. Using a magnet to collect immunoprecipitates on the Dynabeads, supernatant was decanted and beads were washed in 1 ml NENT-150 (20 mM Tris, 150 mM NaCl, 0.5% NP40, 0.1 mM EDTA) and 1 ml wash buffer (50 mM Tris, 150 mM NaCl), and resuspended in 30 μl of 2 × Laemmli sample buffer. Samples were placed in boiling water bath for 5 minutes and centrifuged for 1 min at 10,000 rpm. The supernatant was decanted and 15 µl aliquots were loaded to 4–20% gradient gels for SDS-PAGE. The gel was applied to Whatman paper, treated under vacuum for 2 h at 80 °C and exposed to a phosphor screen. Radiolabeled proteins were detected on a Typhoon imager and data were processed by ImageQuant software.

### Polysome analysis

Cells were transfected for 24 h with indicated molecular clone in two 100 mm plates and then treated with 100 µg/ml cycloheximide (CHX) for 5 min. Cells were harvested in 1 ml PBS/CHX by pipeting, pelleted, and the PBS/CHX was removed. Cells were resuspended in 450 µl ice cold low salt buffer (20 mM Tris-HCl pH7.5, 3 mM MgCl_2_, 10 mM NaCl, 2 mM DTT, Protease inhibitor, 100 µg/ml cycloheximide, 5 µl/ml RNase Out) and placed on ice for 5 min, diluted in 500 µl lysis buffer (0.2 M sucrose, 1.2% Triton X-100, low salt buffer) and treated by ten strokes of a Dounce homogenizer. Following centrifugation to retain cell debris, typically ~1200 µl samples were collected. Eight-percent was extracted with Trizol to ascertain equivalent input RNA. After reserving an aliquot of the lysate, 900 µl was loaded to 13 ml continuous sucrose gradients (10–50%) and centrifuged in an SW41 rotor for 2 h at 25,000 rpm. The Brandel system that collects from the top of the gradient generated twenty-four 0.5 ml fractions. Every other fraction was stored or treated with warm ethanol and 3 M NaOAc overnight to collect nucleic acid. After centrifugation, 100 µl DEPC-treated water was used to resuspend each pellet and the samples were extracted by Trizol and chloroform and precipitated in isopropanol. This pellet was washed with 75% ethanol and resuspended in 11 µl of DEPC-treated water and 10 µl was treated with Omniscript RT. Approximately 20% of the cDNA reaction was used for qPCR with HIV-specific primers, as above. The value of 1/CT was assessed against plasmid standard curves to determine relative copy number per sample. Gradients were evaluated to ensure similar total abundance of gag RNA and the distribution of the transcripts was evaluated on the basis of percentage.

### Statistical analysis

To assess differences in ^35^S-Gag de novo synthesis between each molecular clone and WT over time, we fit a linear mixed model on the log of the ^35^S-Gag phosphor imager (PI) units with time, sample and their interaction as fixed effects and the replicate and the molecular clone within replicate as random effects. For each time point separately and when averaged over the time points, pairwise comparisons between each molecular clone and WT were computed, and p-values corrected for multiple comparisons using Dunnett’s method. Differences ± standard errors were computed on the log scale and back-transformed to ratios. As a control, a similar model was fit using both ^35^S-Gag and ^35^S-beta-Catenin with additional terms for the protein and interactions with protein and time and sample.

To assess differences in proportion of gag transcript between the molecular clones for each RNP, ANOVA models were fit on the log of the proportion, and pairwise comparisons between each molecular clone and WT were computed, with p-values again corrected for multiple comparisons using Dunnett’s method. Data are available upon request.

## Electronic supplementary material

Supplementary information
