# Supplementary material for: The basal translation rate of authentic HIV-1 RNA is regulated by 5’UTR nt-pairings at junction of R and U5
Source: Sci Rep. 2017 Jul 31;7:6902. doi: 10.1038/s41598-017-06883-9 (PMC5537239; doi:10.1038/s41598-017-06883-9)
Supplement: Supplementary file 1 — Supplementary information [file 41598_2017_6883_MOESM1_ESM.pdf]

The basal translation rate of authentic HIV-1 RNA is regulated by 5'UTR nt-pairings at junction of R and U5

Boeras, I[1]., Seufzer, B.[1], Brady, S.[2], Rendahl, A.[1], Heng, X.[2]*, and K. Boris-Lawrie[1]*

* Corresponding authors.

[1] University of Minnesota, Dept of Veterinary and Biomedical Sciences, 1971 Commonwealth, Saint Paul, MN 55108 USA

[2]University of Missouri, Dept of Biochemistry, 503 S. College Ave, Columbia, MO 65211 USA

Supplementary figure legends

Figure S1. Flowchart of process to measure translation rate.

De novo translation activity of mRNA templates was measured by the incorporation of radiolabeled amino acids into nascent polypeptides at 3 consecutive intervals. Gag and beta-Catenin were collected by IP and analyzed by SDS-PAGE. Radiolabeled protein was quantified by phosphorimaging. Drawing made by Ioana Boeras and Kathleen Boris-Lawrie.

Figure S2. Entire gel of the metabolic labeling assay in Fig. 2. SDS-PAGE and phosphorimager analysis of ^{35}S-labeled immunoprecipitated Gag polypeptides (Gag-Pol, Gag precursor, and processed capsid). Beta-Catenin was co-precipitated with exception of the SubB U103C sample, as designated by asterisk. M indicates the lane containing the molecular weight markers (KDa).

Figure S3. Six representative ribosomal RNA profiles. Continuous sucrose gradients of cytoplasmic extracts of HEK293 cells were prepared and distribution of ribosomal RNA was monitored at 254nm.

Figure S4. Monomer-dimer equilibrium assay of WT and U103C 5'UTRs.

In vitro transcribed RNAs corresponding to HIV 5'UTR (+1 to 356) were incubated in physiologic ionic strength buffer to equilibrium and visualized by native agarose electrophoresis.

Table S1. Rate of amino acid incorporation to Gag polypeptide is sensitive to U103 substitution in monomer-prone 5'UTR mutants [a].

Molecular clone [Predominant conformer] [b]	Gag fold difference relative to WT (Average ± standard error)	p-value	beta-Catenin fold difference relative to WT (Average ± standard error)	p-value
WT [Dimer]	1.0	Baseline	1.0	Baseline
SubC [Dimer]	1.1 ± 0.2	0.9319	2.8 ± 0.9	0.0096
SubA [Monomer]	0.7 ± 0.2	0.5592	0.8 ± 0.2	0.9439
SubB [Monomer]	0.8 ± 0.2	0.7829	1.1 ± 0.3	0.9861
SubA U103G [Monomer]	3.1 ± 0.6	0.0001	1.9 ± 0.6	0.1715
SubB U103C [Monomer]	2.6 ± 1.0	0.0541	NA	NA

[a] Average fold difference (± standard error) of de novo Gag and beta-Catenin synthesis over the three time points for each molecular clone relative to WT. Results of three to ten independent experiments were back-transformed from the linear mixed model and standard error was computed by the delta method; p-values adjusted for multiple comparisons by Dunnett's method.

[b] Predominant conformation observed in monomer:dimer equilibrium assay (Figure 1).

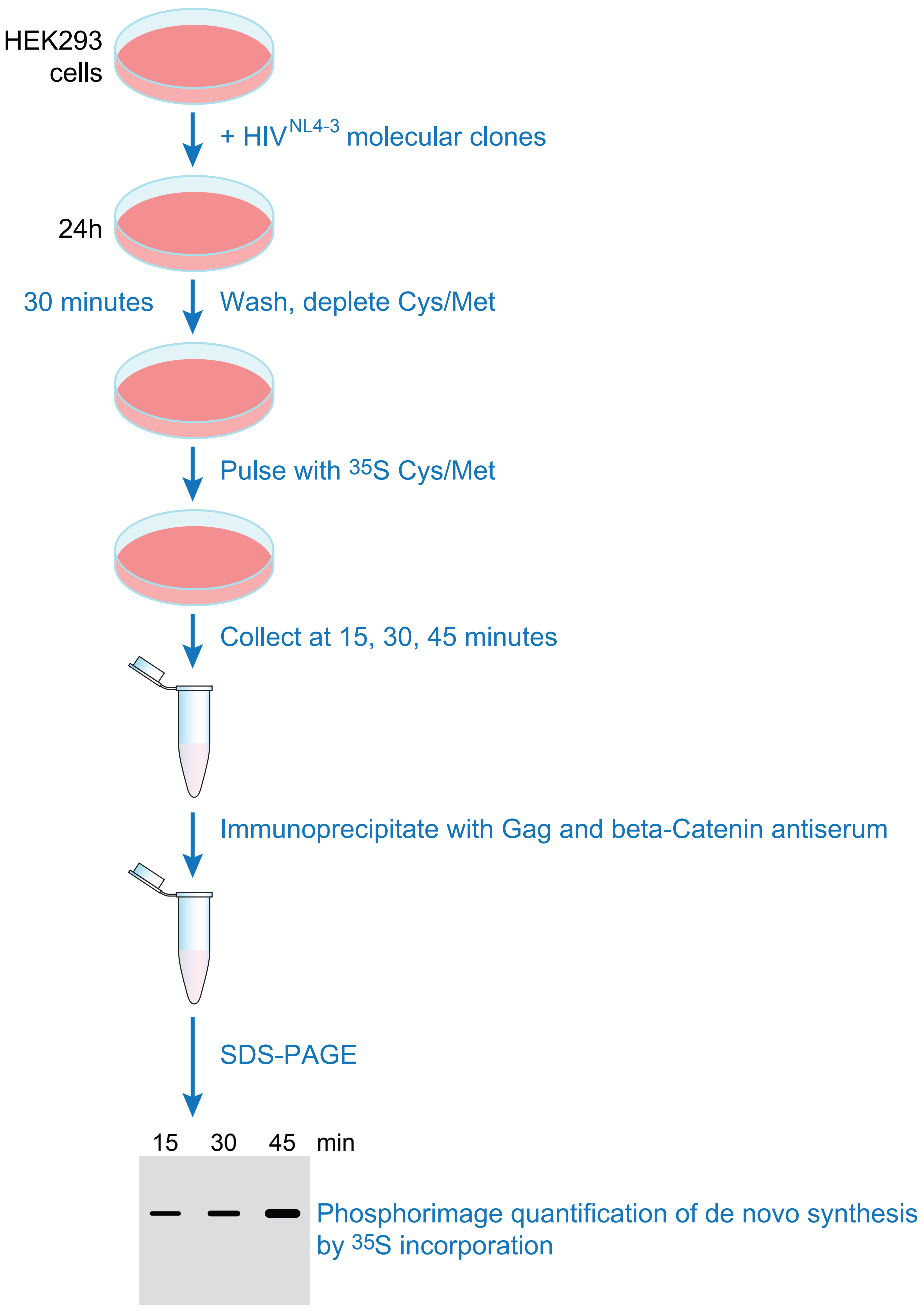
HEK293 cells
+ HIV^{NL4-3} molecular clones
24h
30 minutes
Wash, deplete Cys/Met
Pulse with ^{35}S Cys/Met
Collect at 15, 30, 45 minutes
Immunoprecipitate with Gag and beta-Catenin antiserum
SDS-PAGE
15 30 45 min
Phosphorimage quantification of de novo synthesis by ^{35}S incorporation

Figure S1

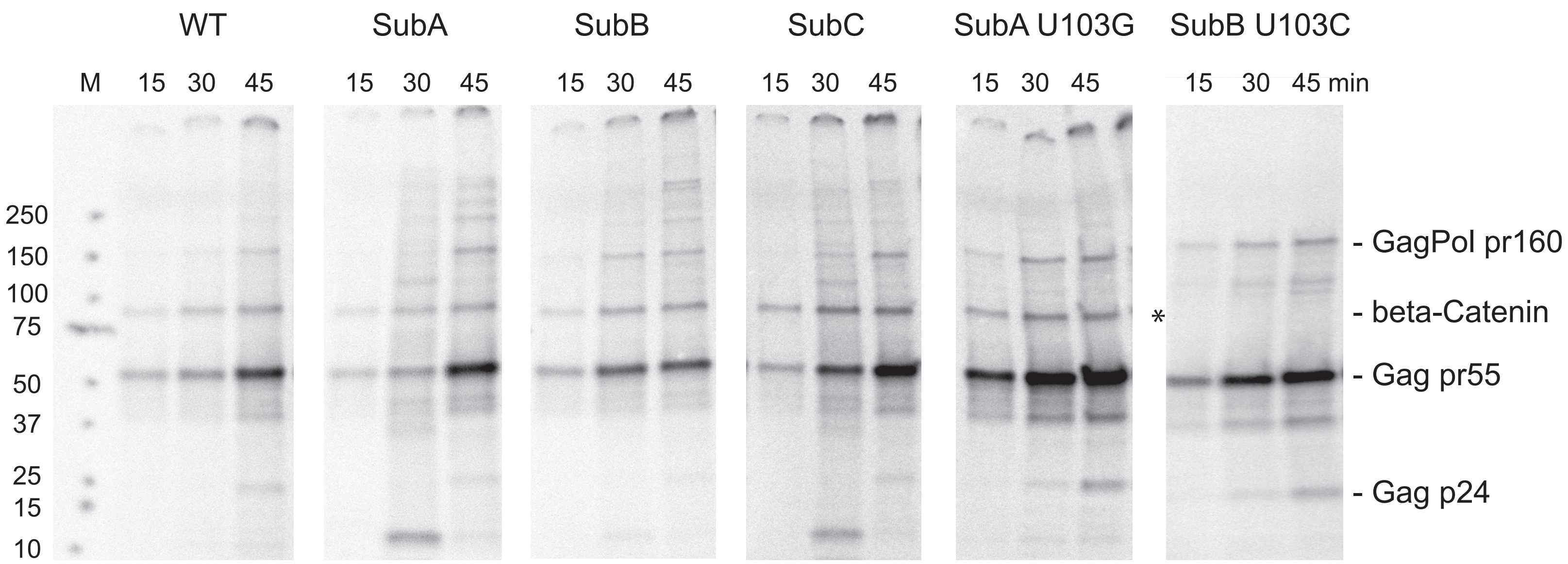
WT
SubA
SubB
SubC
SubA U103G
SubB U103C
M
15
30
45
15
30
45
15
30
45
15
30
45
15
30
45
15
30
45 min
250
150
100
75
50
37
25
15
10
*
- GagPol pr160
- beta-Catenin
- Gag pr55
- Gag p24

Figure S2

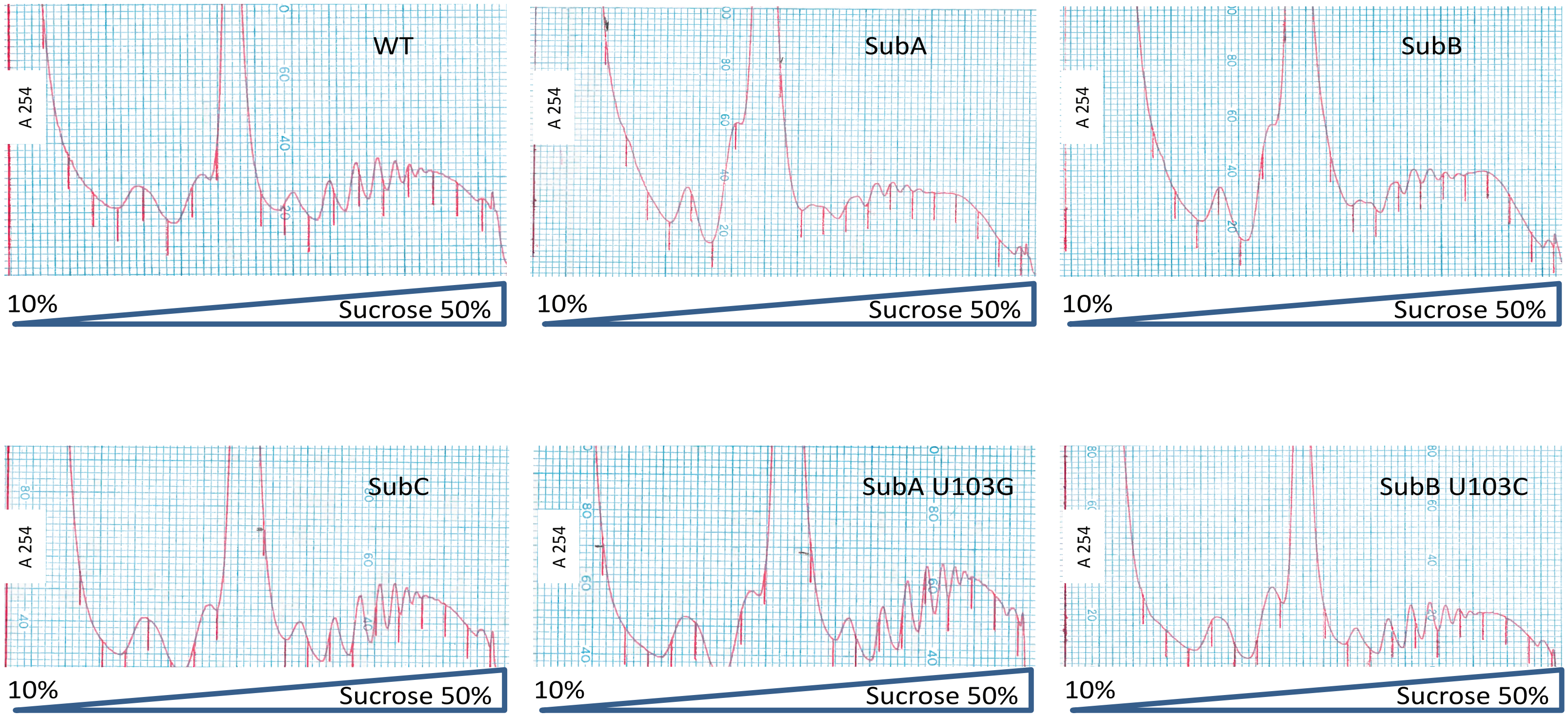

Figure S3

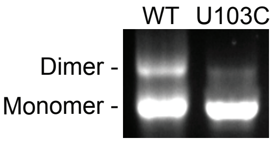

Figure S4
